# The approximation of bivariate Chlodowsky-Szász-Kantorovich-Charlier-type operators

**DOI:** 10.1186/s13660-017-1465-1

**Published:** 2017-08-23

**Authors:** Purshottam Narain Agrawal, Behar Baxhaku, Ruchi Chauhan

**Affiliations:** 1 Department of Mathematics, Indian Institute of Technology Roorkee, Roorkee, 247667 India; 2 Department of Mathematics, University of Prishtina, Mother Teresa, Prishtina, 10000 Kosovo

**Keywords:** GBS operators, Chlodowsky and Szász-type operators, Charlier polynomials, partial moduli of continuity, modulus of smoothness, degree of approximation

## Abstract

In this paper, we introduce a bivariate Kantorovich variant of combination of Szász and Chlodowsky operators based on Charlier polynomials. Then, we study local approximation properties for these operators. Also, we estimate the approximation order in terms of Peetre’s K-functional and partial moduli of continuity. Furthermore, we introduce the associated GBS-case (Generalized Boolean Sum) of these operators and study the degree of approximation by means of the Lipschitz class of Bögel continuous functions. Finally, we present some graphical examples to illustrate the rate of convergence of the operators under consideration.

## Introduction

In [1], Varma and Taşdelen constituted a link between orthogonal polynomials and positive linear operators. They considered Szász-type operators including Charlier polynomials. The generating functions for these Charlier polynomials are given by

1 $$ e^{t} \biggl(1-\frac{t}{a} \biggr)^{u} = \sum_{k=0}^{\infty} \sum _{s=0}^{k}\binom{k}{s}(-u)_{s} \biggl( \frac{1}{a} \biggr)^{s} \frac {t^{k}}{k!} = \sum_{k=0}^{\infty} C_{k}^{a}(u) \frac{t^{k}}{k!}, \quad|t|< a, $$

where $(m)_{0}=1$ and $(m)_{j}=\prod_{k=1}^{j} (m+k-1)$ ($j\in\mathbb{N}$).

The Charlier polynomials $C_{k}^{a}(u)$ for $k=0,1,2,3,4$ are given by

$$\begin{gathered}C_{0}^{a}(u)=1,\qquad C_{1}^{a}(u)=1- \frac{u}{a},\qquad C_{2}^{a}(u)=1-\frac {u(1+2a)}{a^{2}}+ \frac{u^{2}}{a^{2}}, \\ C_{3}^{a}(u)=1-\frac{u}{a^{3}}\bigl(3a^{2}+3a+2 \bigr)+\frac{3u^{2}}{a^{3}}(a+1)-\frac {u^{3}}{a^{3}}, \\ C_{4}^{a}(u)=1-\frac{2u}{a^{4}}\bigl(3+4a+3a^{2}+2a^{3} \bigr)+\frac {u^{2}}{a^{4}}\bigl(11+12a+6a^{2}\bigr)-\frac{2u^{3}}{a^{4}}(3+2a)+ \frac{u^{4}}{a^{4}}. \end{gathered}$$

Further, from equation (1) we note that $C_{k}^{a}(u)>0$ for all $u\leq0$ and $k=0,1,2,\ldots$ .

Varma and Taşdelen defined the following Szász-type operators involving Charlier polynomials

$$L_{n}(f;x,a)=\sum_{k=0}^{\infty}\Pi_{n,k}(nx,a)f \biggl( \frac{k}{n} \biggr), $$

where

$$ \begin{aligned}\Pi_{n,k}(nx,a) &= e^{-1} \biggl(1-\frac{1}{a} \biggr)^{(a-1)nx}\frac {C_{k}^{(a)}(-(a-1)nx)}{k!} \\ &=e^{-1} \biggl(1-\frac{1}{a} \biggr)^{(a-1)nx}\sum _{s=0}^{k}{k \choose s} \bigl((a-1)nx\bigr)_{s} \biggl(\frac{1}{a} \biggr)^{s}>0, \quad a>1, x\in[0,\infty). \end{aligned}$$

Kajla and Agrawal [2–4] discussed some generalizations of Szász-type operators based on Charlier polynomials and obtained some direct results such as Voronovskaja-type asymptotic theorem, weighted approximation properties, and approximation of functions having derivatives of bounded variation. For a detailed account of such kind of results for different types of sequences of linear positive operators and their linear combinations, we refer the readers to a recent book [5].

The classical Bernstein-Chlodowsky polynomials are defined as

$$C_{n}(f;x) = \sum_{k=0}^{n} p_{n,k} \biggl( \frac{x}{a_{n}} \biggr)f \biggl( \frac{k}{n}a_{n} \biggr), $$

where $p_{n,k} ( \frac{x}{a_{n}} ) = \binom{n}{k} ( \frac {x}{a_{n}} )^{k} ( 1-\frac{x}{a_{n}} )^{n-k}$, $0\leq x \leq a_{n}$, and $(a_{n})$ is a sequence of positive numbers with $\lim_{n \to \infty} a_{n} = \infty$ and $\lim_{n \to\infty}\frac{a_{n}}{n} = 0$. There are many investigations devoted to the problem of approximating continuous functions by classical Bernstein-Chlodowsky polynomials and their generalization.

Agrawal and Ispir in [6] introduced the variant of Szász variant-based Charlier polynomials defined as

$$S_{m} (f;x,a)=\sum_{j=0}^{\infty}\Pi_{m,j}(b_{m}y,a)f \biggl(\frac{j}{c_{m}} \biggr), \quad a>1, $$

where $(b_{m})$ and $(c_{m})$ are increasing sequences of positive numbers such that $c_{m}\geq1$, $b_{m} \geq1$, $\lim_{n\to\infty} (1/c_{m}) = 0$, and $b_{m}/c_{m} = 1+ O(1/c_{m})$. Also, Agrawal and Ispir [6] introduced bivariate operators by combining the Bernstein-Chlodowsky operators and Szász-Charlier-type operators as follows:

2 $$ S_{n,m}^{a} (f;x,y) = \sum _{k=0}^{n} \sum_{j=0}^{\infty}p_{n,k} \biggl( \frac{x}{a_{n}} \biggr) \Pi_{m,j}(b_{m}y,a) f \biggl( \frac{k}{n}a_{n}, \frac{j}{c_{n}} \biggr) $$

for all $n,m\in N$, $f\in C(I_{a_{n}}) $ with $I_{a_{n}}= \{(x,y):0\leq x\leq a_{n}, y\geq0 \}$ and $C(I_{a_{n}}) = \{f:I_{a_{n}}\to R^{+}\text{ is continuous}\}$. The weighted approximation properties of bivariate modified Szász operators are studied in [7–9]. Note that the operator $S_{n,m}^{a}$ is the tensorial product of $_{x}C_{n}$ and $_{y}S_{m}^{a}$, that is, $S_{n,m}^{a} = {}_{x}C_{n} \times{}_{y}S_{m}^{a} $, where

$$_{x}C_{n} (f;x,y) = \sum_{k=0}^{n} p_{n,k} \biggl( \frac{x}{a_{n}} \biggr)f \biggl( \frac{ka_{n}}{n}, y \biggr) $$

and

$$_{y}S_{m}^{a} (f;x,y) = \sum _{j=0}^{\infty} \Pi_{m,j}(b_{m}y,a)f \biggl( x, \frac {j}{c_{m}} \biggr). $$

In [7], the authors introduced a bivariate Kantorovich variant of the combination of Chlodowsky and Szász-type operators and studied local approximation properties of these operators. Also, they estimated the approximation order in terms of Peetre’s *K*-functional and partial moduli of continuity.

The rest of the paper is as follows. In Section 2, we construct the bivariate Chlodowsky-Szász-Kantorovich-Charlier-type operators and the convergence of these operators given by means of Korovkin’s theorem. Further, some graphical examples to illustrate the rate of convergence of the operators under consideration are presented. In Section 3, the order of approximation is obtained with help of the partial moduli and continuity and Peetre’s *K*-functional. In Section 4, we study some convergence properties of these operators in weighted spaces with weighted norm on $R_{+}^{2}$ by using the weighted Korovkin-type theorems. In the last section of the paper, we introduce the associated GBS-case (Generalized Boolean Sum) of these operators and study the degree of approximation by means of the Lipschitz class of Bögel continuous functions.

## The construction of the operators

Our goal is to introduce a new bivariate operators associated with a combination of Kantorovich variant of the operators given by (2) as follows: For all $n,m\in N$ and $f\in C(I_{a_{n}})$, we define

3 $$ C_{n,m}^{a}(f;x,y) = \frac{n}{a_{n}}c_{m} \sum_{k=0}^{n} \sum _{j=0}^{\infty}p_{n,k} \biggl(\frac{x}{a_{n}} \biggr) \Pi_{m,j}(b_{m}y,a) \int_{\frac {j}{c_{m}}}^{\frac{j+1}{c_{m}}} \int_{\frac{k}{n}a_{n}}^{\frac{k+1}{n}a_{n}} f(t,s) \,dt\,ds, $$

where $a>1$, and the sequences $(a_{n})$, $(b_{m})$, and $(c_{m}) $ are defined as before and satisfy the following conditions:

4 $$ \lim_{n\to\infty} (a_{n}/n) = 0\quad \text{and}\quad \lim_{m\to\infty} (1/c_{m}) = 0,\qquad b_{m}/c_{m} = 1+ O(1/c_{m}). $$

For operators defined by (3), we have

$$C_{n,m}^{a} (f;x,y) = {}_{x}C^{*}_{n} \bigl(_{y}^{*}S_{m}^{a}(f;x,y)\bigr) ={}_{y}^{*}S_{m}^{a} \bigl(_{x}C^{*}_{n}(f;x,y) \bigr), $$

where

$$_{x}C^{*}_{n}(f;x,y) = \frac{n}{a_{n}}\sum _{k=0}^{n} p_{n,k} \biggl( \frac {x}{a_{n}} \biggr) \int_{\frac{k}{n}a_{n}}^{\frac{k+1}{n}a_{n}}f(t,y)\,dt $$

and

$$_{y}^{*}S^{a}_{m}(f;x,y) = c_{m} \sum _{j=0}^{\infty}\Pi_{m,j}(b_{m}y,a) \int_{\frac {j}{c_{m}}}^{\frac{j+1}{c_{m}}}f(x,s)\,ds. $$

Next, the degree of approximation of the operator $C_{n,m}^{a} $ given by (3) will be established in the space of continuous function on the compact set $I_{de} = [0,d]\times[0,e] \subset I_{a_{n}}$. For $I_{de} = [0,d]\times[0,e]$, let $C(I_{de})$, denote the space of all real-valued continuous functions on $I_{de}$, endowed with the norm $\| f \|_{C(I_{de})} = \sup_{(x,y)\in I_{de}}|f(x,y)|$. In what follows, let $e_{ij}:I_{a_{n}}\to R$, $e_{ij}(x,y) = x^{i}y^{j}$, $(x,y)\in I_{a_{n}}$, $(i,j)\in N^{0} \times N^{0} $ with $i+j \leq4 $, be the two-dimensional test functions. In the following, we give some lemmas. We observe that there are some slips in the calculation of the moments in Lemma 1 of [6]. We give correct values in the following lemma.

### Lemma 2.1

*For the operators* $_{x}C^{*}_{n}$ *and* $_{y}^{*}S^{a}_{m}$, *we have the following inequalities*:

(i) ${}_{x}C^{*}_{n}(e_{00};x,y)=1$;
(ii) ${}_{x}C^{*}_{n}(e_{10};x,y)=x$;
(iii) ${}_{x}C^{*}_{n}(e_{20};x,y)= (1-\frac{1}{n} )x^{2}+\frac {a_{n}x}{n}$;
(iv) ${}_{y}^{*}S^{a}_{m}(e_{01};x,y)=\frac{b_{m}y+1}{c_{m}}$;
(v) ${}_{y}^{*}S^{a}_{m}(e_{02};x,y)=\frac{b_{m}^{2}y^{2}}{c_{m}^{2}}+\frac {b_{m}}{c_{m}^{2}} (2+\frac{1}{(a-1)} )y+\frac{1}{c_{m}^{2}}$;
(vi) ${}_{y}^{*}S^{a}_{m}(e_{03};x,y)=\frac{b_{m}^{3}y^{3}}{c_{m}^{3}}+\frac {3b_{m}^{2}}{c_{m}^{3}} (1+\frac{1}{(a-1)} )y^{2}+\frac{b_{m}}{c_{m}^{3}} (3+\frac{3}{(a-1)}+\frac{2}{(a-1)^{2}} )y+\frac{1}{c_{m}^{3}}$;
(vii) ${}_{y}^{*}S^{a}_{m}(e_{04};x,y)=\frac{b_{m}^{4}y^{4}}{c_{m}^{4}}+\frac {2b_{m}^{3}}{c_{m}^{3}} (1+\frac{1}{(a-1)} )y^{3} +\frac{b_{m}^{2}y^{2}}{c_{m}^{4}} (4+\frac{6}{(a-1)}+\frac {11}{(a-1)^{2}} ) +\frac{2b_{m}y}{c_{m}^{4}} (2+\frac{2}{(a-1)}+ \frac{2}{(a-1)^{2}}+\frac {3}{(a-1)^{3}} )+\frac{1}{c_{m}^{4}}$.

### Lemma 2.2

*The following statements hold*;

(i) $C_{n,m}^{a} (e_{00};x,y)=1$;
(ii) $C_{n,m}^{a} (e_{10};x,y)=x+\frac{a_{n}}{2n}$;
(iii) $C_{n,m}^{a} (e_{01};x,y)=\frac{b_{m}y}{c_{m}}+\frac{3}{2c_{m}}$;
(iv) $C_{n,m}^{a} (e_{20};x,y)= ( 1-\frac{1}{n} ) x^{2} +2\frac{a_{n}}{n}x +\frac{a_{n}^{2}}{3n^{2}}$;
(v) $C_{n,m}^{a} (e_{30};x,y)=x^{3}+\frac{3a_{n}x^{2}}{n} (1-\frac {x}{a_{n}} )+\frac{a_{n}^{2}x}{n^{2}} (1-\frac{x}{a_{n}} ) (1-\frac{2x}{a_{n}} ) +\frac{3a_{n}}{2n} (\frac{a_{n}^{2}}{3n^{2}}+ (1-\frac{1}{n} )x^{2}+\frac{2a_{n}x}{n} ) + \frac{a_{n}^{2}}{n^{2}} (x+\frac{a_{n}}{2n} )+\frac{1}{4a_{n}^{3}}$;
(vi) $C_{n,m}^{a}(e_{40};x,y)=x^{4}+6x^{3}\frac{a_{n}}{n} (1-\frac {x}{a_{n}} )+\frac{a_{n}^{2}x^{2}}{n^{2}} (6 (1-\frac{x}{a_{n}^{2}} )^{2} -3\frac{x}{a_{n}} (1-\frac{x}{a_{n}} )+ (1-\frac{x}{a_{n}} ) (1-\frac{2x}{a_{n}} ) )+\frac{x a_{n}^{3}}{n^{3}} ( (1-\frac{x}{a_{n}} )^{2} (1-\frac{2x}{a_{n}} ) -\frac{x}{a_{n}} (1-\frac{x}{a_{n}} ) (1-\frac{2x}{a_{n}} )-\frac{2x}{a_{n}} (1-\frac{x}{a_{n}} )^{2} ) + \frac{2a_{n}}{n} (x^{3}+\frac{3a_{n}x^{2}}{n} (1-\frac{x}{a_{n}} )+\frac{a_{n}^{2}x}{n^{2}} (1-\frac{x}{a_{n}} ) (1-\frac {2x}{a_{n}} ) +\frac{3a_{n}}{2n} (\frac{a_{n}^{2}}{3n^{2}}+ (1-\frac{1}{n} )x^{2}+\frac{2a_{n}x}{n} )+ \frac{a_{n}^{2}}{n^{2}} (x+\frac {a_{n}}{2n} )+\frac{1}{4a_{n}^{3}} ) +\frac{2a_{n}^{2}}{n^{2}} (\frac{a_{n}^{2}}{3n^{2}}+ (1-\frac{1}{n} )x^{2}+\frac{2a_{n}x}{n} )+\frac{a_{n}^{3}}{n^{3}} (x+\frac {a_{n}}{2n} )+\frac{a_{n}^{4}}{5n^{4}}$;
(vii) $C_{n,m}^{a} (e_{01};x,y)=\frac{b_{m}y}{c_{m}}+\frac{3}{2c_{m}}$;
(viii) $C_{n,m}^{a} (e_{02};x,y)=\frac{b_{m}^{2}y^{2}}{c_{m}^{2}}+\frac {b_{m}y}{c_{m}^{2}} (3+\frac{1}{(a-1)} )+\frac{7}{3c_{m}^{2}}$;
(ix) $C_{n,m}^{a} (e_{03};x,y)=\frac{b_{m}^{3}y^{3}}{c_{m}^{3}}+\frac {b_{m}^{2}y^{2}}{c_{m}^{3}} (\frac{9}{2}+\frac{3}{a-1} )+\frac {b_{m}y}{c_{m}^{3}} ( 7+\frac{9}{2(a-1)}+\frac{2}{(a-1)^{2}} ) +\frac {15}{4c_{m}^{3}} $;
(x) $C_{n,m}^{a} (e_{04};x,y)=\frac{b_{m}^{4}y^{4}}{c_{m}^{4}}+\frac {b_{m}^{3}y^{3}}{c_{m}^{4}} ( 4+\frac{6}{a-1} ) +\frac {b_{m}^{2}y^{2}}{c_{m}^{4}} (12+\frac{12}{a-1}+\frac{11}{(a-1)^{2}} ) +\frac{b_{m}y}{c_{m}^{4}} ( 15+\frac{12}{a-1}+\frac{8}{(a-1)^{2}}+ \frac {6}{(a-1)^{3}} ) +\frac{31}{5c_{m}^{4}}$.

### Proof

By Lemma 2.1 we have

$$\begin{aligned} C_{n,m}^{a}(e_{00};x,y)&=\frac{nc_{m}}{a_{n}}\sum _{k=0}^{n}\sum_{j=0}^{\infty}P_{n,k} \biggl(\frac{x}{a_{n}} \biggr)\Pi_{m,j}(b_{m}y,a) \int_{\frac{j}{c_{m}}}^{\frac{j+1}{c_{m}}} \int_{\frac{k a_{n}}{n}}^{\frac {(k+1)a_{n}}{n}}1\,dt\,ds \\ &=\sum_{k=0}^{n}\sum _{j=0}^{\infty}P_{n,k} \biggl(\frac{x}{a_{n}} \biggr)\Pi _{m,j}(b_{m}y,a)=1 \end{aligned}$$

and

$$\begin{aligned} C_{n,m}^{a}(e_{10};x,y)&=\frac{nc_{m}}{a_{n}}\sum _{k=0}^{n}\sum_{j=0}^{\infty}P_{n,k} \biggl(\frac{x}{a_{n}} \biggr)\Pi_{m,j}(b_{m}y,a) \int_{\frac{j}{c_{m}}}^{\frac{j+1}{c_{m}}} \int_{\frac{k a_{n}}{n}}^{\frac {(k+1)a_{n}}{n}}t \,dt\,ds \\ &=\frac{n}{a_{n}}\sum_{k=0}^{n}\sum _{j=0}^{\infty}P_{n,k} \biggl( \frac {x}{a_{n}} \biggr)\Pi_{m,j}(b_{m}y,a) \biggl( \frac{1}{2} \biggl(\frac{a_{n}}{n} \biggr)^{2}+ \frac{k a_{n}^{2}}{n^{2}} \biggr) \\ &=\frac{a_{n}}{2n}+x. \end{aligned}$$

Again by Lemma 2.1

$$\begin{aligned}& \begin{aligned} C_{n,m}^{a}(e_{01};x,y)&=\frac{nc_{m}}{a_{n}}\sum _{k=0}^{n}\sum_{j=0}^{\infty}P_{n,k} \biggl(\frac{x}{a_{n}} \biggr)\Pi_{m,j}(b_{m}y,a) \int_{\frac{j}{c_{m}}}^{\frac{j+1}{c_{m}}} \int_{\frac{k a_{n}}{n}}^{\frac {(k+1)a_{n}}{n}}s \,dt\,ds \\ &=c_{m}\sum_{k=0}^{n}\sum _{j=0}^{\infty}P_{n,k} \biggl( \frac {x}{a_{n}} \biggr)\Pi_{m,j}(b_{m}y,a) \biggl( \frac{1}{2} \biggl(\frac{1}{c_{m}} \biggr)^{2}+ \frac{j}{c_{m}^{2}} \biggr) \\ &=\frac{3}{2c_{m}}+\frac{b_{m}y}{c_{m}}, \end{aligned}\\& \begin{aligned} C_{n,m}^{a}(e_{20};x,y)&=\frac{nc_{m}}{a_{n}}\sum _{k=0}^{n}\sum_{j=0}^{\infty}P_{n,k} \biggl(\frac{x}{a_{n}} \biggr)\Pi_{m,j}(b_{m}y,a) \int_{\frac{j}{c_{m}}}^{\frac{j+1}{c_{m}}} \int_{\frac{k a_{n}}{n}}^{\frac {(k+1)a_{n}}{n}}t^{2} \,dt\, ds \\ &=\frac{n}{a_{n}}\sum_{k=0}^{n}\sum _{j=0}^{\infty}P_{n,k} \biggl( \frac {x}{a_{n}} \biggr)\Pi_{m,j}(b_{m}y,a) \biggl( \frac{a_{n}^{3}}{3n^{3}}+\frac{k^{2}a_{n}^{3}}{n^{3}}+\frac{k a_{n}^{3}}{n^{3}} \biggr) \\ &=\frac{a_{n}^{2}}{3n^{2}}+ \biggl(1-\frac{1}{n} \biggr)x^{2}+ \frac{2a_{n}x}{n}, \end{aligned} \end{aligned}$$

and

$$ \begin{aligned}C_{n,m}^{a}(e_{30};x,y)={}&\frac{nc_{m}}{a_{n}}\sum _{k=0}^{n}\sum_{j=0}^{\infty}P_{n,k} \biggl(\frac{x}{a_{n}} \biggr)\Pi_{m,j}(b_{m}y,a) \int_{\frac{j}{c_{m}}}^{\frac{j+1}{c_{m}}} \int_{\frac{k a_{n}}{n}}^{\frac {(k+1)a_{n}}{n}}t^{3} \,dt\, ds \\ ={}&\sum_{k=0}^{n}\sum _{j=0}^{\infty}P_{n,k} \biggl(\frac{x}{a_{n}} \biggr)\Pi_{m,j}(b_{m}y,a) \biggl(\frac{k^{3}a_{n}^{3}}{n^{3}}+ \frac{3k^{2}a_{n}^{3}}{2n^{3}}+\frac{k a_{n}^{3}}{n^{3}}+\frac{a_{n}^{3}}{4n^{3}} \biggr) \\ ={}&x^{3}+\frac{3a_{n}x^{2}}{n} \biggl(1-\frac{x}{a_{n}} \biggr)+ \frac {a_{n}^{2}x}{n^{2}} \biggl(1-\frac{x}{a_{n}} \biggr) \biggl(1- \frac{2x}{a_{n}} \biggr) \\ &+\frac{3a_{n}}{2n} \biggl(\frac{a_{n}^{2}}{3n^{2}}+ \biggl(1-\frac{1}{n} \biggr)x^{2}+\frac{2a_{n}x}{n} \biggr)+\frac{a_{n}^{2}}{n^{2}} \biggl(x+\frac {a_{n}}{2n} \biggr)+\frac{1}{4a_{n}^{3}}.\end{aligned} $$

Further,

$$\begin{aligned} C_{n,m}^{a}(e_{40};x,y)={}&\frac{nc_{m}}{a_{n}}\sum _{k=0}^{n}\sum_{j=0}^{\infty}P_{n,k} \biggl(\frac{x}{a_{n}} \biggr)\Pi_{m,j}(b_{m}y,a) \int_{\frac{j}{c_{m}}}^{\frac{j+1}{c_{m}}} \int_{\frac{k a_{n}}{n}}^{\frac {(k+1)a_{n}}{n}}t^{4} \,dt\, ds \\ ={}&\sum_{k=0}^{n}\sum _{j=0}^{\infty}P_{n,k} \biggl(\frac{x}{a_{n}} \biggr)\\ &\times\Pi_{m,j}(b_{m}y,a) \biggl(\frac{k^{4}a_{n}^{4}}{n^{4}}+ \frac{2k^{3}a_{n}^{4}}{n^{4}}+\frac {2k^{2}a_{n}^{4}}{n^{4}}+\frac{k a_{n}^{4}}{n^{4}}+\frac{a_{n}^{4}}{5n^{4}} \biggr) \\ ={}&x^{4}+6x^{3}\frac{a_{n}}{n} \biggl(1- \frac{x}{a_{n}} \biggr)\\ &+\frac {a_{n}^{2}x^{2}}{n^{2}} \biggl(6 \biggl(1- \frac{x}{a_{n}^{2}} \biggr)^{2} -3\frac{x}{a_{n}} \biggl(1- \frac{x}{a_{n}} \biggr)+ \biggl(1-\frac{x}{a_{n}} \biggr) \biggl(1- \frac{2x}{a_{n}} \biggr) \biggr) \\ &+\frac{x a_{n}^{3}}{n^{3}} \biggl( \biggl(1-\frac{x}{a_{n}} \biggr)^{2} \biggl(1-\frac{2x}{a_{n}} \biggr)\\ & -\frac{x}{a_{n}} \biggl(1- \frac{x}{a_{n}} \biggr) \biggl(1-\frac{2x}{a_{n}} \biggr)-\frac{2x}{a_{n}} \biggl(1-\frac{x}{a_{n}} \biggr)^{2} \biggr) \\ &+\frac{2a_{n}}{n} \biggl(x^{3}+\frac{3a_{n}x^{2}}{n} \biggl(1- \frac{x}{a_{n}} \biggr)+\frac{a_{n}^{2}x}{n^{2}} \biggl(1-\frac{x}{a_{n}} \biggr) \biggl(1-\frac {2x}{a_{n}} \biggr) \\ &+\frac{3a_{n}}{2n} \biggl(\frac{a_{n}^{2}}{3n^{2}}+ \biggl(1-\frac{1}{n} \biggr)x^{2}+\frac{2a_{n}x}{n} \biggr)+\frac{a_{n}^{2}}{n^{2}} \biggl(x+ \frac {a_{n}}{2n} \biggr)+\frac{1}{4a_{n}^{3}} \biggr) \\ &+\frac{2a_{n}^{2}}{n^{2}} \biggl(\frac{a_{n}^{2}}{3n^{2}}+ \biggl(1-\frac {1}{n} \biggr)x^{2}+\frac{2a_{n}x}{n} \biggr)+\frac{a_{n}^{3}}{n^{3}} \biggl(x+ \frac {a_{n}}{2n} \biggr)+\frac{a_{n}^{4}}{5n^{4}} \end{aligned}$$

and

$$ \begin{aligned}C_{n,m}^{a}(e_{02};x,y)&=\frac{nc_{m}}{a_{n}}\sum _{k=0}^{n}\sum_{j=0}^{\infty}P_{n,k} \biggl(\frac{x}{a_{n}} \biggr)\Pi_{m,j}(b_{m}y,a) \int_{\frac{j}{c_{m}}}^{\frac{j+1}{c_{m}}} \int_{\frac{k a_{n}}{n}}^{\frac {(k+1)a_{n}}{n}}s^{2} \,dt\, ds \\ &=\sum_{k=0}^{n}\sum _{j=0}^{\infty}P_{n,k} \biggl(\frac{x}{a_{n}} \biggr)\Pi_{m,j}(b_{m}y,a) \biggl(\frac{j}{3c_{m}^{2}}+ \frac{j^{2}}{c_{m}^{2}}+\frac{j}{c_{m}^{2}} \biggr) \\ &=\frac{b_{m}^{2}y^{2}}{c_{m}^{2}}+\frac{b_{m}y}{c_{m}^{2}} \biggl(3+\frac{1}{(a-1)} \biggr)+ \frac{7}{3c_{m}^{2}}. \end{aligned}$$

Again by Lemma 2.1

$$\begin{aligned} C_{n,m}^{a}(e_{03};x,y)&=\frac{nc_{m}}{a_{n}}\sum _{k=0}^{n}\sum_{j=0}^{\infty}P_{n,k} \biggl(\frac{x}{a_{n}} \biggr)\Pi_{m,j}(b_{m}y,a) \int_{\frac{j}{c_{m}}}^{\frac{j+1}{c_{m}}} \int_{\frac{k a_{n}}{n}}^{\frac {(k+1)a_{n}}{n}}s^{3} \,dt\, ds \\ &=\sum_{k=0}^{n}\sum _{j=0}^{\infty}P_{n,k} \biggl(\frac{x}{a_{n}} \biggr)\Pi_{m,j}(b_{m}y,a) \biggl(\frac{j^{3}}{c_{m}^{3}}+ \frac{3j^{2}}{2c_{m}^{3}}+\frac{j}{c_{m}^{4}}+\frac {1}{c_{m}^{4}} \biggr) \\ &=\frac{b_{m}^{3}y^{3}}{c_{m}^{3}}+\frac{b_{m}^{2}y^{2}}{c_{m}^{3}} \biggl(\frac {9}{2}+ \frac{3}{a-1} \biggr)+\frac{b_{m}y}{c_{m}^{3}} \biggl( 7+\frac {9}{2(a-1)}+ \frac{2}{(a-1)^{2}} \biggr) +\frac{15}{4c_{m}^{3}} \end{aligned}$$

and

$$\begin{aligned} C_{n,m}^{a}(e_{04};x,y)={}&\frac{nc_{m}}{a_{n}}\sum _{k=0}^{n}\sum_{j=0}^{\infty}P_{n,k} \biggl(\frac{x}{a_{n}} \biggr)\Pi_{m,j}(b_{m}y,a) \int_{\frac{j}{c_{m}}}^{\frac{j+1}{c_{m}}} \int_{\frac{k a_{n}}{n}}^{\frac {(k+1)a_{n}}{n}}s^{4} \,dt\, ds \\ ={}&\sum_{k=0}^{n}\sum _{j=0}^{\infty}P_{n,k} \biggl(\frac{x}{a_{n}} \biggr)\Pi_{m,j}(b_{m}y,a) \biggl(\frac{j^{4}}{c_{m}^{4}}+ \frac{2j^{3}}{c_{m}^{4}}+\frac {2j^{2}}{c_{m}^{4}}+\frac{j}{c_{m}^{4}}+\frac{1}{5c_{m}^{4}} \biggr) \\ ={}&\frac{b_{m}^{4}y^{4}}{c_{m}^{4}}+\frac{b_{m}^{3}y^{3}}{c_{m}^{4}} \biggl( 4+\frac{6}{a-1} \biggr) + \frac{b_{m}^{2}y^{2}}{c_{m}^{4}} \biggl(12+\frac{12}{a-1}+\frac {11}{(a-1)^{2}} \biggr) \\ &+\frac{b_{m}y}{c_{m}^{4}} \biggl( 15+\frac {12}{a-1}+\frac{8}{(a-1)^{2}}+ \frac{6}{(a-1)^{3}} \biggr) +\frac{31}{5c_{m}^{4}}. \end{aligned}$$

 □

### Remark 2.3

By applying Lemma 2.2 we have

$$\begin{gathered} C_{n,m}^{a} \bigl( (e_{10}-x)^{2};x,y \bigr) = \frac {x(a_{n}-x)}{n} + \frac{a_{n}^{2}}{3n^{2}} ; \\ C_{n,m}^{a} \bigl( (e_{01}-y)^{2};x,y \bigr) = \biggl( \frac{b_{m}}{c_{m}} - 1 \biggr)^{2} y^{2} + \biggl( \frac{b_{m}}{c_{m}^{2}} \biggl(4+\frac{1}{a-1} \biggr) - \frac{3}{c_{m}} \biggr) y + \frac{10}{3c_{m}^{2}}. \end{gathered} $$

Hence, for all $(x,y) \in I_{a_{n}}$ and sufficiently large *n*, *m*, by Lemma 2.2, Remark 2.3, and condition (4) we can write

5 $$\begin{aligned}& C_{n,m}^{a} \bigl( (e_{10}-x)^{2};x,y \bigr) = O \biggl( \frac{a_{n}}{n} \biggr) \Biggl(\sum _{i=0}^{2}x^{i}\Biggr), \end{aligned}$$

6 $$\begin{aligned}& C_{n,m}^{a} \bigl( (e_{10}-x)^{4};x,y \bigr) = O \biggl( \frac{a_{n}}{n} \biggr) \Biggl(\sum _{i=0}^{4}x^{i} \Biggr), \end{aligned}$$

7 $$\begin{aligned}& C_{n,m}^{a} \bigl( (e_{10}-y)^{2};x,y \bigr) \leq\frac{\tau(a)}{c_{m}} \Biggl(\sum_{i=0}^{2}y^{i} \Biggr), \end{aligned}$$

and

8 $$ C_{n,m}^{a} \bigl( (e_{10}-y)^{4};x,y \bigr) \leq\frac{\omega(a)}{c_{m}} \Biggl(\sum_{i=0}^{4}y^{i} \Biggr), $$

where $\tau(a)$ and $\omega(a)$ are constants depending on $a>1$. For $(x,y)\in I_{de}$, by relations (5) and (7) we may write

9 $$\begin{aligned}& C_{n,m}^{a} \bigl( (e_{10}-x)^{2};x,y \bigr) \leq\frac{a_{n}(x^{2}+x)}{n}+\frac {a_{n}^{2}}{3n^{2}} \leq\frac{a_{n}(d^{2}+d)}{n}+ \frac{a_{n}^{2}}{n}+\frac {a_{n}^{2}}{3n^{2}} = \rho(d)\frac{a_{n}}{n}, \end{aligned}$$

10 $$\begin{aligned}& C_{n,m}^{a} \bigl( (e_{10}-y)^{2};x,y \bigr) \leq\frac{\tau (a)}{c_{m}}\bigl(y^{2}+y+1\bigr) \leq \frac{\tau(a)}{c_{m}}\bigl(b^{2}+b+1\bigr)=\frac{\gamma(a)}{c_{m}}, \end{aligned}$$

where $\rho(d)$ is a constant depending on *d*, and $\gamma(a)$ is a constant depending on ${a>1}$. Further, let $\delta_{n}(x) = C_{n,m}^{a} ( (e_{10}-x)^{2};x,y )$, $\delta_{m}(y) = C_{n,m}^{a} ( (e_{01}-y)^{2};x,y )$, and $\delta_{n,m}(x,y) = ( O ( \frac {a_{n}}{n} ) (\sum_{i=0}^{2}x^{i}) +\frac{\tau(a)}{c_{m}}(\sum_{i=0}^{2}y^{i}) )^{1/2}$.

### Definition 2.1

See [10]

For $f\in C(I_{de})$ and $\delta>0$, the complete modulus of continuity for the function $f(x,y)$ is defined by

$$\omega(f;\delta_{n}, \delta_{m}) = \sup\bigl\{ \big|f(t,s)-f(x,y)\big|:(t,s),(x,y) \in I_{de}, |t-x|\leq\delta_{n}, |s-y|\leq\delta_{m} \bigr\} , $$

and its partial modulus of continuity with respect to *x* and *y* is given by

$$\begin{gathered} \omega^{(1)}(f;\delta) = \sup_{0\leq y \leq e} \sup _{|x_{1}-x_{2}| \leq \delta} \bigl\{ \big|f(x_{1}, y) - f(x_{2}, y) \big| \bigr\} , \\\omega^{(2)}(f;\delta) = \sup_{0\leq x \leq d} \sup _{|y_{1}-y_{2}| \leq \delta} \bigl\{ \big|f(x, y_{1}) - f(x, y_{2}) \big| \bigr\} . \end{gathered}$$

### Definition 2.2

(See [11]) For $f\in C(I_{de}) $ and $\delta>0$, the Peetre’s *K*-functional and the second modulus of smoothness are defined respectively as

$$K(f; \delta) = \inf_{g\in C^{2}(I_{de})} \bigl\{ {\|} f-g {\| }_{C(I_{de})}+ \delta {\|}g {\|}_{C^{2}(I_{de})} \bigr\} $$

and

$$\omega_{2} (f;\delta) = \sup_{\sqrt{t^{2}+s^{2}}\leq\delta} \big\| \Delta _{t,s}^{2}f(x,y) \big\| , $$

where $\Delta_{t,s}^{2}f(x,y)=\sum_{j=0}^{2}(-1)^{2-j} \binom {2}{j}f(x+jt,y+js)$. Here, $C^{2}(I_{de})$ is the space of functions *f* such that $\frac{\partial^{i} f}{\partial x^{i}}, \frac{\partial^{i} f}{\partial y^{i}} \in C(I_{de})$ ($i=1,2$). The norm on the space $C^{2}(I_{de}) $ is defined as

$$\| f \|_{C^{2}(I_{de})} = \| f \|_{C(I_{de})}+\sum _{i=1}^{2} \biggl( {\bigg\| }\frac{\partial^{i} f}{\partial x^{i}} { \bigg\| }_{C_{(I_{de})}} + {\bigg\| }\frac{\partial^{i} f}{\partial y^{i}} {\bigg\| }_{C_{(I_{de})}} \biggr). $$

It is known that ([12], p.192) there exists a positive constant, independent of *δ* and *f*, such that

$$K(f; \delta) \leq L \bigl\{ \omega_{2} (f;\delta) +\min(1, \delta) \Vert f \Vert _{C(I_{de})} \bigr\} . $$

To study the convergence of the sequence $\{ C_{n,m}^{a} (f;x, y) \} $, we shall use the following Korovkin-type theorem established by Volkov.

### Theorem 2.4

*If* $f\in C(I_{de})$, *then the operators* $C_{n,m}^{a}$ *given by* (3) *converge uniformly to* *f* *on the compact set* $I_{de}$ *as* $n,m \to \infty$.

### Proof

By Lemma 2.2, taking into account the equality (3), we find

$$\lim_{n,m\to\infty} {\big\| } C_{n,m}^{a}(e_{ij};x,y) - e_{ij} {\big\| }_{C(I_{de})} = 0,\quad i,j=0,1,2, $$

and

$$\lim_{n,m\to\infty} {\big\| } C_{n,m}^{a}(e_{20}+e_{02};x,y) - e_{20} + e_{02} {\big\| }_{C(I_{de})} = 0. $$

The proof of uniform convergence is then completed by applying the Volkov theorem [13]. □

### Example 1

For $n = m = 50$ and $a_{n} = \sqrt{n}$, $b_{m} = m$, $c_{m} = m+\frac{1}{\sqrt{m}}$, in Table 1, we have estimated the absolute difference between the operators $C_{n,m}^{a}(f;x,y)$ defined in (2) and the function $f(x,y)=xe^{-x^{2}}y^{2} e^{-y}$. Also, we have estimated the absolute difference between the operators $L^{*}_{n,m}(f;x,y)$ defined by Ispir and Buyukyazici [7] and the function $f(x,y)=xe^{-x^{2}}y^{2} e^{-y}$.

**Table 1:** **Error of approximation for** $\pmb{C_{n,m}^{a}}$ **and** $\pmb{L^{*}_{n,m}}$

**(** ***x*** **,** ***y*** **)**	**(0.01,0.01)**	**(0.01,0.11)**	**(0.01,0.21)**	**(0.01,0.31)**	**(0.01,0.41)**	**(0.01,0.51)**
$|C_{n,m}^{a}(f;x,y)-f(x,y)|$	0.000011	0.000022	0.000032	0.000043	0.000054	0.000064
$|L^{*}_{n,m}(f;x,y)-f(x,y)|$	0.000036	0.000039	0.000043	0.000046	0.000049	0.000052

**Figure 1:**
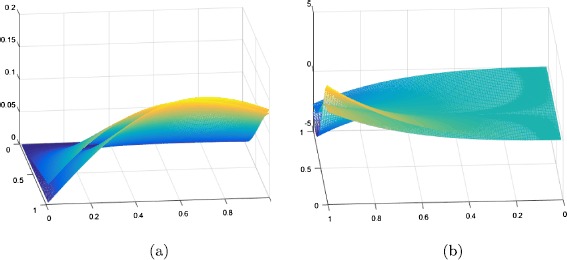
**The convergence of the operators** $\pmb{C^{a}_{n;m}(f; x; y)}$ **to function** $\pmb{f(x; y)}$ **.**

For $n = m = 50$ and $a_{n} = \sqrt{n}$, $b_{m} = m$, $c_{m} = m+\frac{1}{\sqrt {m}}$ the convergence of bivariate Chlodowsky-Szász-Kantorovich-Charlier-type operators $C_{n,m}^{a}(f;x,y) $ to the function $f(x,y)=xe^{-x^{2}}y^{2} e^{-y}$ is illustrated in Figure 1(a). In Table 2, we have estimated the absolute difference between the operators $C_{n,m}^{a}(f;x,y)$ defined in (3) and the function $f(x,y)=xe^{-x^{2}}y^{2} e^{-y}$. Also, we have estimated the absolute difference between the operators $S^{*}_{n,m}(f;x,y)$ defined in [6] and the function $f(x,y)=xe^{-x^{2}}y^{2} e^{-y}$. It easily can be seen from Table 2 that the absolute difference $|C_{n,m}^{a}(f;x,y) - f(x,y)|<|S_{n,m}^{a}(f;x,y) - f(x,y)|$. Thus, the rate of convergence of the operators $C_{n,m}^{a}$ to the function is faster compared to the operators defined in [14].

**Table 2:** **Error of approximation for** $\pmb{C_{n,m}^{a}}$ **and** $\pmb{S^{a}_{n,m}}$

**(** ***x*** **,** ***y*** **)**	**(0.01,0.01)**	**(0.01,0.11)**	**(0.01,0.21)**	**(0.01,0.31)**	**(0.01,0.41)**	**(0.01,0.51)**
$|C_{n,m}^{a}(f;x,y)-f(x,y)|$	0.000011	0.000022	0.000032	0.000043	0.000054	0.000064
$|S^{a}_{n,m}(f;x,y)-f(x,y)|$	0.000137	0.000152	0.000167	0.000181	0.000196	0.000210

### Example 2

Let us consider the function $f:R^{2} \to R$, $f(x,y) = x^{2} e^{x}\cos(\pi y)$. For $n=m = 50$ and $a = 2$, $a_{n}=\sqrt{n}$, $b_{m} = m$, $c_{m}=m+\frac{1}{\sqrt {m}} $, the convergence of bivariate Chlodowsky-Szász-Kantorovich-Charlier-type operators $C_{n,m}^{a}(f;x,y) $ to the function $f(x,y)$ is illustrated in Figure 1(b).

## Degree of approximation

Now, we obtain the rate of convergence of the approximation of the bivariate operators defined in (3) by means of modulus of continuity of functions.

### Theorem 3.1

*For any* $f\in C(I_{de})$, *we have the following inequalities*:

$$ \big| C_{n,m}^{a} (f;x, y) - f(x,y) \big| \leq2 \bigl( \omega^{(1)}(f; \delta_{n} ) +\omega^{(2)}(f; \delta_{m} ) \bigr) $$

*and*

$$ \big| C_{n,m}^{a} (f;x, y) - f(x,y)\big| \leq2 \omega(f; \delta_{n,m} ), $$

*where* $\delta_{n} = \delta_{n}(x)$, $\delta_{m} = \delta_{m}(y)$, *and* $\delta _{n,m} = \delta_{n,m}(x,y)$.

### Proof

From (3) by using Lemma 2.2 and the definition of partial moduli of continuity of a function $f(x,y)$ we can write

$$\begin{gathered} \big| C_{n,m}^{a} (f;x, y) - f(x,y) \big| \leq C_{n,m}^{a} \bigl( \big|f(t,s) -f(x,y)\big|;x,y \bigr) \\ \quad \leq C_{n,m}^{a} \bigl( \big|f(t,s) -f(x,s)\big|;x,y \bigr) + C_{n,m}^{a} \bigl( \big|f(x,s) -f(x,y)\big|;x,y \bigr) \\ \quad\leq C_{n,m}^{a} \bigl( \omega^{(1)}\bigl(f; |t-x|\bigr);x,y \bigr) + C_{n,m}^{a} \bigl( \omega^{(2)}\bigl(f; |s-y|\bigr);x,y \bigr) \\ \quad\leq\omega^{(1)}(f; \delta_{n}) \bigl( 1+{ \delta_{n}^{-1}} C_{n,m}^{a}\bigl(|t-x|;x,y\bigr) \bigr) + \omega^{(2)}(f; \delta_{m}) \bigl( 1+{ \delta_{m}^{-1}} C_{n,m}^{a}\bigl(|s-y|;x,y\bigr) \bigr). \end{gathered}$$

Then, by the Cauchy-Schwarz inequality we have

$$\begin{aligned} \big| C_{n,m}^{a} (f;x, y) - f(x,y) \big|\leq{}&\omega^{(1)}(f; \delta_{n}) \biggl( 1+\frac{1}{\delta_{n}} \bigl\{ C_{n,m}^{a} \bigl((e_{10}-x)^{2};x,y\bigr) \bigr\} ^{1/2} \biggr) \\ &+ \omega^{(2)}(f; \delta_{m}) \biggl( 1+ \frac {1}{\delta_{n}} \bigl\{ C_{n,m}^{a}\bigl((e_{01}-y)^{2};x,y \bigr) \bigr\} ^{1/2} \biggr). \end{aligned}$$

Finally, choosing $\delta_{n} = \delta_{n}(x)$ and $\delta_{m} = \delta _{m}(x)$, we reach the desired result for all $(x,y) \in I_{de}$.

To prove the second part of this theorem, we will use relations (4) and (6) and well-known properties of the modulus of continuity. Thus, we have

$$\begin{aligned} \big| C_{n,m}^{a} (f;x, y) - f(x,y) \big| &\leq C_{n,m}^{a} \bigl( \omega \bigl(f; \sqrt{(t-x)^{2}+(s-y)^{2}}; x,y\bigr) \bigr) \\ &\leq\omega(f; \delta_{n,m}) \biggl( 1+\frac{1}{\delta_{n,m}} C_{n,m}^{a}\bigl(\sqrt{(t-x)^{2}+(s-y)^{2}};x,y \bigr) \biggr). \end{aligned}$$

Recalling the Cauchy-Schwarz inequality, we obtain

$$\begin{aligned} \big| C_{n,m}^{a} (f;x, y) - f(x,y) \big| &\leq \omega(f; \delta_{n,m}) \biggl( 1+\frac{1}{\delta_{n,m}} \bigl( C_{n,m}^{a} \bigl({(t-x)^{2}+(s-y)^{2}};x,y\bigr) \bigr)^{1/2} \biggr) \\ &\leq\omega(f; \delta_{n,m}) \Biggl( 1+\frac{1}{\delta_{n,m}} \Biggl( O \biggl( \frac{a_{n}}{n} \biggr) \Biggl(\sum_{i=0}^{2}x^{i} \Biggr) + \frac{\tau (a)}{c_{m}}\Biggl(\sum_{i=0}^{2}y^{i} \Biggr) \Biggr)^{1/2} \Biggr). \end{aligned}$$

Taking $\delta_{n,m} = ( O ( \frac{a_{n}}{n} ) (\sum_{i=0}^{2}x^{i}) + \frac{\tau(a)}{c_{m}}(\sum_{i=0}^{2}y^{i}) )^{1/2}$, we obtain the desired result. In what follows, we introduce the Lipschitz class in the bivariate case. For $0<\gamma_{1} \leq1$ and $0<\gamma_{2} \leq1$, we define the Lipschitz class

$$\operatorname{Lip}_{L} (f;\gamma_{1}, \gamma_{2}) = \bigl\{ f:\big|f(t,s) - f(x,y) \big| \leq L |t-x|^{\gamma_{1}}|s-y|^{\gamma_{2}} \bigr\} , $$

where $(t,s), (x,y)\in I_{de}$. □

### Theorem 3.2

*Suppose that* $f\in \operatorname{Lip}_{L} (f;\gamma_{1}, \gamma_{2})$. *Then*, *for every* $(x,y)\in I_{de}$, *we have*

$$\big| C_{n,m}^{a} (f;x, y) - f(x,y) \big| \leq L( \delta_{n})^{\gamma _{1}/2}(\delta_{m})^{\gamma_{2}/2}, $$

*where* $\delta_{n}=\delta_{n}(x)$ *and* $\delta_{m}=\delta_{m}(y)$.

### Proof

Taking into account that $f\in \operatorname{Lip}_{L}(f;\gamma_{1}, \gamma _{2})$ and using the monotonicity and linearity of operators $C_{n,m}^{a} (f;x, y) $, we have

$$\begin{aligned} \big| C_{n,m}^{a} (f;x, y) - f(x,y) \big| &\leq C_{n,m}^{a} \bigl(\big|f(t,s)-f(x,y)\big|;x,y\bigr) \\ &\leq L C_{n,m}^{a} \bigl( |t-x|^{\gamma_{1}} |s-y|^{\gamma_{2}}; x, y \bigr) \\ &\leq L \cdot{}_{x}C_{n}^{*} (\vert t-x|^{\gamma_{1}};x,y ) _{y}^{*} S_{m}^{a} \bigl( |s-y|^{\gamma _{2}};x,y \bigr). \end{aligned}$$

For $({u}_{1},{v}_{1})= (\frac{2}{\gamma_{1}},\frac{2}{1-\gamma _{1}} ) $ and $({u}_{2},{v}_{2})= (\frac{2}{\gamma_{2}},\frac {2}{1-\gamma_{2}} )$, applying the Hölder inequality, we get

$$\begin{aligned} \big| C_{n,m}^{a} (f;x, y) - f(x,y)\big| &\leq L \bigl(_{x}C_{n}^{*}\bigl((t-x)^{2};x;y\bigr) \bigr)^{{\gamma_{1}/2}} \bigl(_{y}S_{n}^{*} (s-y)^{2};x;y \bigr)^{ {\gamma_{2}/2}} \\ &\leq(\delta_{n})^{\gamma_{1}/2}(\delta_{m})^{\gamma_{2}/2}, \end{aligned}$$

which implies the desired result. □

### Theorem 3.3

*Let* $f \in C^{1}(I_{de})$. *Then*, *for every* $(x,y) \in I_{de}$, *we have the inequality*

$$\bigl\vert C_{n, m}^{a} (f; x, y) -f(x, y) \bigr\vert \leq\big\| f_{x}' \big\| _{C(I_{de})} \sqrt{ \delta_{n}(x)}+\big\| f'_{y}\big\| _{C(I_{de})} \sqrt {\delta_{m}(y)}. $$

### Proof

For a fixed point $(x,y) \in I_{de}$ and for $f \in C^{1}(I_{de})$, we obtain

$$f(u,v) - f(x,y) = \int_{x}^{u} f_{t}'(t,v)\,dt + \int_{y}^{v} f_{z}'(x,z)\, dz \quad\text{for } (u,v)\in I_{de}. $$

Applying the operator defined in (3) to both sides, we obtain

$$C_{n, m}^{a}\bigl(f(u,v); x, y\bigr) -f(x, y) = C_{n, m}^{a} \biggl( \int_{x}^{u} f_{t}'(t,v) \,dt; x, y \biggr) +C_{n, m}^{a} \biggl( \int_{y}^{v} f_{z}'(x, z) \,dz; x, y \biggr). $$

Now, using the sup-norm on $I_{de}$, we get

$$\biggl\vert \int_{x}^{u} f_{t}'(t,v)\,dt \biggr\vert \leq \int_{x}^{u} \bigl\vert f_{t}'(t,v) \,dt \bigr\vert |du| \leq\big\| f'_{x} \big\| _{C_{(I_{de})}} |u-x| $$

and

$$\biggl\vert \int_{y}^{v} f_{z}' (x,z) \,dz \biggr\vert \leq \int_{y}^{v} \bigl\vert f_{z}'(x,z) \bigr\vert |\,dz| \leq\big\| f_{y}'\big\| _{C_{(I_{de})}} |v-y|. $$

By using these inequalities we have

11 $$\begin{aligned}[b] &\bigl\vert C_{n, m}^{a} \bigl(f(u,v); x, y \bigr) -f(x, y) \bigr\vert \\&\quad\leq C_{n, m}^{a} \biggl( \biggl\vert \int_{x}^{u} f_{t}' (t,v) \,dt \biggr\vert ; x, y \biggr)+C_{n, m}^{a} \biggl( \biggl\vert \int_{y}^{v} f_{z}' (x,z) \,dz \biggr\vert ; x, y \biggr) \\ &\quad\leq\big\| f_{x}' \big\| _{C(I_{de})} C_{n, m}^{a}\bigl(|u-x|; x, y\bigr) + \big\| f_{y}' \big\| _{C(I_{de})} C_{n, m}^{a} \bigl(|v-y|; x, y\bigr). \end{aligned}$$

Now, applying the Hölder inequality, the equality $C_{n, m}^{a} (1; x, y)=1$, and Remark 2.3, we get

12 $$ C_{n, m}^{a} \bigl(|u-x|; x, y\bigr) \leq \bigl\{ _{x}C_{n}^{*}\bigl((u-x)^{2}; x, y\bigr) \times _{x}C_{n}^{*}(1; x, y) \bigr\} ^{1/2} \leq \bigl\{ \delta_{n}(x) \bigr\} ^{1/2}. $$

Analogously,

13 $$ C_{n, m}^{a} \bigl(|v-y|; x, y\bigr) \leq \bigl\{ _{y}^{*}S_{m}^{a}\bigl((v-y)^{2};x ,y \bigr) \times _{y}^{*}S_{m}^{a}(1; x, y) \bigr\} ^{1/2} \leq \bigl\{ \delta_{m}(y) \bigr\} ^{1/2}. $$

Combining equations (11)-(13), we obtain

$$\bigl\vert C_{n, m}^{a} (f; x, y) -f(x, y) \bigr\vert \leq \big\| f'_{x} \big\| _{C(I_{de})} \sqrt{ \delta_{n}(x)} + \big\| f'_{y} \big\| _{C(I_{de})} \sqrt{ \delta_{m}(y)}. $$

This completes the proof. □

### Theorem 3.4

*Let* $f\in C(I_{de})$. *Consider the operators*

14 $$ \hat{C}_{n, m}^{a} (f; x, y) = C_{n, m}^{a} (f; x, y) +f(x,y) - f \biggl(x+ \frac{a_{n}}{2n},\frac{b_{m}y}{c_{m}}+\frac{3}{2c_{m}} \biggr). $$

*Then*, *for all* $g\in C^{2} (I_{de})$, *we have the estimate* $C_{n, m}^{a} (f; x, y) -f(x,y) \leq L \{ \omega_{2} ( f; \sqrt {\chi_{n,m}(x, y)} ) + \min \{ 1, \chi_{n,m }( x, y) \} \| f \|_{C(I_{de})} \} +\omega ( f; \sqrt{(\frac {a_{n}}{2n})^{2}+(\frac{b_{m}y}{c_{m}}+\frac{3}{2c_{m}}-y)^{2}} )$, *where* $\chi_{n,m }( x, y)=O ( \frac{a_{n}}{n} ) (x^{2}+x+1)+(\frac {a_{n}}{2n})^{2}+\frac{\tau(a)}{c_{m}} (y^{2}+y+1)+\frac{ ((b_{m}-c_{m})y+3 )^{2}}{c_{m}^{2}}$.

### Proof

From (14) by Lemma 2.2 we have $\hat {C}_{n, m}^{a}(1;x,y)=1$, $\hat{C}_{n, m}^{a} (u-x; x, y) =0$, and $\hat{C}_{n, m}^{a} (v-y; x, y) =0$. By Taylor’s expansion for $g \in C^{2}(I_{de})$, we may write

15 $$\begin{aligned}[b] g(u,v)-g(x,y) ={}& \frac{\partial g(x,y)}{\partial x}(u-x) + \int _{x}^{u}(u-\eta) \frac{\partial^{2} g(\eta, y)}{\partial\eta^{2}}\,d\eta \\ &+ \frac{\partial g(x,y)}{\partial y} (v-y) + \int_{y}^{v}(v-\zeta) \frac {\partial^{2} g(x, \zeta)}{\partial\zeta^{2}}\,d\zeta, \end{aligned}$$

and applying the operators $\hat{C}_{n, m}^{a} (f; x, y) $ to both sides of the equality and using Lemma 2.2, we obtain

$$\begin{aligned} \hat{C}_{n, m}^{a} \bigl(g(u,v); x, y\bigr) - \hat{C}_{n, m}^{a}\bigl(g(x,y)\bigr) = {}&\hat {C}_{n, m}^{a} \biggl( \int_{x}^{u} (u-\eta) \frac{\partial^{2} g(\eta, y)}{\partial\eta^{2}} \,d\eta; x, y \biggr) \\ &+ \hat{C}_{n, m}^{a} \biggl( \int_{y}^{v} (v-\zeta) \frac{\partial^{2} g(x, \zeta)}{\partial\zeta^{2}} \,d\zeta; x, y\biggr) \\ ={}& {C}_{n, m}^{a} \biggl( \int_{x}^{u} (u-\eta) \frac{\partial^{2} g(\eta, y)}{\partial\eta^{2}} \,d\eta; x, y\biggr)\\ & - \int_{x}^{x+\frac{a_{n}}{2n}} \biggl(x+\frac{a_{n}}{2n}-\eta \biggr) \frac{\partial^{2} g(x, \eta)}{\partial\eta^{2}} \,d\eta \\ &+ {C}_{n, m}^{a} \biggl( \int_{y}^{v} (v-\zeta) \frac{\partial^{2} g(\zeta, x)}{\partial\zeta^{2}} \,d\zeta; x, y\biggr)\\ & - \int_{y}^{\frac{b_{m}y}{c_{m}}+\frac{3}{2c_{m}}} \biggl(\frac {b_{m}y}{c_{m}}+ \frac{3}{2c_{m}}-\zeta\biggr) \frac{\partial^{2} g(x, \zeta )}{\partial\zeta^{2}} \,d\zeta. \end{aligned}$$

On the other hand, since

$$\begin{aligned} \bigg\vert \int_{x}^{u} (u-\eta) \frac{\partial^{2} g(\eta, y)}{\partial\eta ^{2}} \,d\eta \bigg\vert & \leq \bigg\vert \int_{x}^{u} \big|(u-\eta)\big| \bigg\vert \frac {\partial^{2} g(\eta, y)}{\partial\eta^{2}} \bigg\vert \,d\eta \bigg\vert \\ &\leq\| g \|_{C^{2}(I_{de})} \bigg\vert \int_{x}^{u} |u-\eta| \bigg\vert \frac {\partial^{2} g(\eta, y)}{\partial\eta^{2}} \bigg\vert \,d\eta \bigg\vert \leq \| g \|_{C^{2}(I_{de})} (u-x)^{2}\end{aligned}$$

and

$$\bigg\vert \int_{x}^{x+\frac{a_{n}}{2n}} (x+\frac{a_{n}}{2n}-\eta) \frac {\partial^{2} g(\eta, y)}{\partial\eta^{2}} \,d\eta\bigg\vert \leq\biggl(\frac {a_{n}}{2n}\biggr)^{2} \| g \|_{C^{2}(I_{de})} $$

and, analogously,

$$\bigg\vert \int_{y}^{v} (v-\zeta) \frac{\partial^{2} g(x, \zeta)}{\partial \zeta^{2}} \,d\zeta \bigg\vert \leq \| g \|_{C^{2}(I_{de})} (v-y)^{2}$$

and

$$\bigg\vert \int_{y}^{\frac{b_{m}y}{c_{m}}+\frac{3}{2c_{m}}} \biggl(\frac {b_{m}y}{c_{m}}+\frac{3}{2c_{m}}-\zeta\biggr) \frac{\partial^{2} g(x, \zeta )}{\partial\zeta^{2}} \,d\zeta \bigg\vert \leq\biggl(\frac{b_{m}y}{c_{m}}+\frac {3}{2c_{m}}-y\biggr)^{2} \| g \|_{C^{2}(I_{de})},$$

we conclude that

16 $$\begin{aligned} \bigl\vert \hat{C}_{n, m}^{a} ( g; x, y) - g (x,y) ) \bigr\vert \leq{}&{C}_{n, m}^{a} \biggl( \biggl\vert \int_{x}^{u} (u-\eta) \frac{\partial^{2} g(\eta, y)}{\partial\eta^{2}} \,d\eta \biggr\vert ; x, y\biggr) \\ &+ \biggl\vert \int_{x}^{x+\frac{a_{n}}{2n}} \biggl(x+\frac{a_{n}}{2n}-\eta \biggr) \frac{\partial ^{2} g( x, \eta)}{\partial\eta^{2}} \,d\eta \biggr\vert \\ &+ {C}_{n, m}^{a} \biggl( \biggl\vert \int_{y}^{v} (v-\zeta) \frac{\partial^{2} g(x, \zeta)}{\partial\zeta ^{2}} \,d\zeta \biggr\vert ; x, y \biggr) \\ &+ \biggl\vert \int_{y}^{\frac{b_{m}y}{c_{m}}+\frac{3}{2c_{m}}} \biggl(\frac{b_{m}y}{c_{m}}+ \frac {3}{2c_{m}}-\zeta\biggr) \frac{\partial^{2} g( x, \zeta)}{\partial\zeta^{2}} \,d\zeta \biggr\vert \\ \leq{}& \biggl\{ {}_{x}C_{n}^{*} \bigl( (u-x)^{2}; x, y\bigr) + \biggl(\frac {a_{n}}{2n} \biggr)^{2} \biggr\} \| g\|_{C^{2}_{(I_{de})}} \\ &+ \biggl\{ {}_{y}S_{m}^{*} \bigl( (v-y)^{2}; x, y \bigr) + \biggl(\frac{b_{m}y}{c_{m}}+\frac {3}{2c_{m}}-y \biggr)^{2} \biggr\} \| g\|_{C^{2}_{(I_{de})}} \\ \leq{}& \biggl\{ O \biggl( \frac{a_{n}}{n} \biggr) \bigl(x^{2}+x+1 \bigr)+\biggl(\frac{a_{n}}{2n}\biggr)^{2} \\ &+\frac{\tau (a)}{c_{m}} \bigl(y^{2}+y+1\bigr)+\frac{ ((b_{m}-c_{m})y+3 )^{2}}{c_{m}^{2}} \biggr\} \| g \|_{C^{2}(I_{de})} \\ ={}& \chi_{n, m}(x, y) \| g \|_{C^{2}(I_{de})}. \end{aligned}$$

Additionally, by (3) and (14) and Lemma 2.2 we have

17 $$ \bigl\vert \hat{C}_{n, m}^{a} ( f; x, y ) \bigr\vert \leq \bigl\vert {C}_{n, m}^{a} ( f; x, y ) \bigr\vert + \big|f(x,y)\big| + \biggl\vert f \biggl(x+\frac{a_{n}}{2n}, \frac{b_{m}y}{c_{m}}+\frac{3}{2c_{m}} \biggr) \biggr\vert \leq 3 \| f \|_{C(I_{de})}. $$

Hence, in view of (3) and (16), we have

$$\begin{aligned} \bigl\vert \hat{C}_{n, m}^{a} ( f; x, y ) -f(x,y) \bigr\vert ={}& \biggl\vert \hat{C}_{n, m}^{a} ( f; x, y ) -f(x,y)+f \biggl(x+\frac {a_{n}}{2n}, \frac{b_{m}y}{c_{m}}+\frac{3}{2c_{m}} \biggr) - f(x,y) \biggr\vert \\ \leq {}&\bigl\vert \hat{C}_{n, m}^{a} ( f-g; x, y ) \bigr\vert + \bigl\vert \hat{C}_{n, m}^{a} ( g; x, y ) - g(x,y) \bigr\vert \\ &+ \big|g(x, y) - f(x, y)\big| + \biggl\vert f \biggl(x+\frac{a_{n}}{2n}, \frac{b_{m}y}{c_{m}}+\frac{3}{2c_{m}} \biggr) -f(x, y) \biggr\vert \\ \leq{}&4 \| f-g \|_{C(I_{de})} + \bigl\vert \hat{C}_{n, m}^{a} ( g; x, y ) -g(x,y) \bigr\vert \\ & + \biggl\vert f \biggl(x+\frac{a_{n}}{2n}, \frac{b_{m}y}{c_{m}}+ \frac{3}{2c_{m}} \biggr) - f(x, y) \biggr\vert \\ \leq{}& \bigl( 4 \| f-g \|_{C(I_{ab})} + \chi_{n, n_{2}} (x, y) \bigr) \| g \|_{C(I_{de})} \\ &+\omega \biggl(f; \sqrt{ \biggl(\frac{a_{n}}{2n} \biggr)^{2}+ \biggl(\frac {b_{m}y}{c_{m}}+\frac{3}{2c_{m}}-y \biggr)^{2}} \biggr)\\ \leq{}&4K \bigl( f; \chi _{n, m} ( x, y) \bigr) +\omega \biggl(f; \sqrt{ \biggl(\frac {a_{n}}{2n} \biggr)^{2}+ \biggl(\frac{b_{m}y}{c_{m}}+\frac{3}{2c_{m}}-y \biggr)^{2}} \biggr)\\ \leq{}& L \bigl\{ \omega_{2} \bigl(f; \sqrt{\chi_{n, m} ( x, y)} \bigr) + \min\bigl\{ 1,\chi_{n, m}( x, y) \bigr\} \| f \|_{C_{(I_{de})}} \bigr\} \\ &+ \omega \biggl(f; \sqrt{ \biggl(\frac{a_{n}}{2n} \biggr)^{2}+ \biggl(\frac {b_{m}y}{c_{m}}+\frac{3}{2c_{m}}-y \biggr)^{2}} \biggr). \end{aligned}$$

This completes the proof. □

## Weighted approximation properties

Let $R_{+}^{2} = \{(x,y): x\geq0, y\geq0\}$, and $B_{\rho}(R_{+}^{2})$ be the space of all functions such that $|f(x,y)|\leq M_{f} \rho(x,y)$, where $(x,y)\in R_{+}^{2}$, and $M_{f}$ is a constant depending on a function *f* only. By $C_{\rho}(R_{+}^{2})$ we denote the subspace of all continuous functions belonging to $B_{\rho}(R_{+}^{2})$. It is clear that $C_{\rho}(R_{+}^{2})$ is a linear normed space with the norm $\| f \|_{\rho} = \sup_{(x, y)\in R_{+}^{2}} \frac{|f(x,y)|}{\rho(x,y)}$. Also, let $C_{\rho}^{*}(R_{+}^{2})$ be the subspace of all functions $f\in C_{\rho}(R_{+}^{2})$ for which $\lim_{\sqrt{x^{2}+y^{2}}\to\infty}\frac {f(x,y)}{1+x^{2}+y^{2}}=k_{f}<\infty$.

### Lemma 4.1

[15, 16]

*For the sequence of positive linear operators* $\{ K_{n, m} \}_{n, m\geq1}$ *acting from* $C_{\rho}(R_{+}^{2})$ *to* $B_{\rho}(R_{+}^{2})$, *it is necessary and sufficient that inequality*

$$\big\| K_{n, m}(\rho; x, y) \big\| _{\rho}\leq k $$

*is fulfilled with some positive constant k*.

### Theorem 4.2

[[15, 16]] *If a sequence of positive linear operators* $K_{n, m}$ *acting from* $C_{\rho}(R_{+}^{2})$ *to* $B_{\rho}(R_{+}^{2})$ *satisfies the conditions*

18 $$\begin{aligned}& \lim_{n, m \to\infty} \big\| K_{n, m}(e_{00}; x, y)-1 \big\| _{\rho}= 0, \end{aligned}$$

19 $$\begin{aligned}& \lim_{n, m \to\infty} \big\| K_{n, m}(e_{10}; x, y)-x \big\| _{\rho}= 0, \end{aligned}$$

20 $$\begin{aligned}& \lim_{n, m \to\infty} \big\| K_{n, m}(e_{01}; x, y)-1 \big\| _{\rho}= 0, \end{aligned}$$

21 $$\begin{aligned}& \lim_{n, m \to\infty} \big\| K_{n, m} \bigl((e_{20}+ e_{02}); x, y\bigr)-\bigl(x^{2}+ y^{2}\bigr) \big\| _{\rho}= 0, \end{aligned}$$

*then*, *for any function* $f \in C_{\rho}^{k}(R_{+}^{2})$

$$\lim_{n, m \to\infty} \big\| K_{n, m}f-f \big\| _{\rho}=0, $$

*and there exists a function* $f^{*} \in C_{\rho}(R_{+}^{2})\setminus C_{\rho}^{k}(R_{+}^{2})$ *for which*

$$\lim_{n, m \to\infty} \big\| K_{n, m}f^{*}-f^{*} \big\| _{\rho}\geq1. $$

### Theorem 4.3

[15, 16]

*Let* $K_{n, m}$ *be a sequence of linear operators acting from* $C_{\rho}(R_{+}^{2})$ *to* $B_{\rho}(R_{+}^{2})$, *and let* $\rho_{1}(x, y) \geq1$ *be a continuous function for which*

22 $$ \lim_{|v| \to\infty} \frac{\rho(v)}{\rho_{1}(v)} = 0 \quad\bigl( \textit{where } v = (x,y)\bigr) . $$

*If* $K_{n, m}$ *satisfies the conditions of Theorem* 4.2, *then*

$$\lim_{n, m \to\infty} \| K_{n, m}f-f \|_{\rho_{1}} = 0 $$

*for all* $f \in C_{\rho}(R_{+}^{2})$.

Now, we consider the positive linear operators $K_{n, m}$ defined by

23 $$ K_{n, m}(f; x, y) = \left\{ \textstyle\begin{array}{l@{\quad}l} {C}_{n, m}^{a}(f;x, y) & \mbox{for } (x,y) \in I_{a_{n}d_{m}}, \\ f(x, y) & \mbox{for } (x,y) \in R_{+}^{2}\setminus I_{a_{n}d_{m}}, \end{array}\displaystyle \right. $$

where $I_{a_{n}d_{m}} = \{ (x, y):0\leq x \leq a_{n}, 0\leq y \leq d_{m} \}$, and $(d_{m})$ is a sequence such that $\lim_{m\to\infty }d_{m}=\infty$.

### Theorem 4.4

*Let* $\rho(x,y)=1+x^{2}+y^{2}$ *be a weight function*, *and* $K_{n, m}(f; x, y)$ *be a sequence of linear positive operators defined by* (23). *Then*, *for all* $f\in C_{\rho}(R_{+}^{2})$, *we have*

$$\lim_{n, m \to\infty} \| K_{n, m}f-f \|_{\rho_{1}} = 0, $$

*where* $\rho_{1}(x, y)$ *is a continuous function satisfying condition* (22).

### Proof

First, we show that $K_{n, m}$ is acting from $C_{\rho}(R_{+}^{2})$ to $B_{\rho}(R_{+}^{2})$. Using Lemma 2.2, we can write

$$\begin{aligned} \big\| K_{n, m}(\rho; x,y) \big\| _{\rho}\leq{}&1+ \biggl( 1- \frac{1}{n} \biggr) \sup_{(x,y)\in I_{a_{n} d_{m}}}\frac{x^{2}}{\rho(x,y)} + 2 \frac{a_{n}}{n} \sup_{(x,y)\in I_{a_{n} d_{m}}} \frac{x}{\rho(x,y)} + \frac{a_{n}^{2}}{3n^{2}} \\ &+\frac{b_{m}^{2}}{c_{m}^{2}} \sup_{(x,y)\in I_{a_{n} d_{m}}} \frac{y^{2}}{\rho (x,y)}+ \frac{b_{m}}{c_{m}^{2}}\sup_{(x,y)\in I_{a_{n}d_{m}}} \frac{y}{\rho(x,y)}+ \frac{10}{3c_{m}^{2}} \\ \leq{}&1+ \varphi_{n,m}+\psi_{n,m}, \end{aligned}$$

where $\varphi_{n,m} = ( 1- \frac{1}{n} ) + \frac {b_{m}^{2}}{c_{m}^{2}}$ and $\psi_{n,m} = \frac{b_{m}}{c_{m}^{2}}+2\frac{a_{n}}{n}+\frac {a_{n}^{2}}{3n^{2}}+\frac{10}{3c_{m}^{2}}$. Since $\lim_{n,m\to\infty}\varphi _{n,m} = 2$ and $\lim_{n,m\to\infty}\psi_{n,m} = 0$, there exists a positive constant *k* such that $\varphi_{n,m}+\psi_{n,m} < k$ for all natural numbers *n* and *m*. Hence, we have

$$\big\| K_{n,m}(\rho; x,y)\big\| _{\rho}\leq1+k. $$

From Lemma 4.1 we have $K_{n,m}:C_{\rho}(R_{+}^{2}) \to B_{\rho}(R_{+}^{2})$. If we can show that the conditions of Theorem 4.2 are satisfied, then the proof of Theorem 4.4 is completed. Using Lemma 2.2, we can obtain (18)-(20). Finally, using Lemma 2.2, we get

$$\big\| K_{n,m}(e_{20}+e_{02}; x,y) - \bigl(x^{2}+y^{2}\bigr) \big\| _{\rho}\leq \psi_{n,m}+\frac {1}{n}+\bigg|\frac{b_{m}^{2}}{c_{m}^{2}}-1\bigg|= \eta_{n,m}, $$

and since $\lim_{n,m\to\infty} \eta_{n,m} =0$, we obtain the desired result. □

### Theorem 4.5

*Let* $\{K_{n, m}\}$ *be a sequence linear positive operators defined by* (23). *Then*, *for each function* $f\in C_{\rho}(R_{+}^{2})$, *we have*

$$\lim_{n, m \to\infty} \| K_{n, m}f-f \|_{\rho}=0. $$

### Proof

From (18)-(21) we have

$$\lim_{n,m\to\infty} {\big\| } K_{n,m}^{a}(e_{ij};x,y) - e_{ij} {\big\| }_{\rho} = 0, \quad i,j\in\{0,1\}, $$

and

$$\lim_{n,m\to\infty} {\big\| } K_{n,m}^{a}(e_{20}+e_{02};x,y) - (e_{20} + e_{02}) {\big\| }_{\rho} = 0, $$

and using Theorem 4.2, we obtain the desired result.

Now we compute the order of approximation of the operators $C_{n,m}^{a}$ in terms of the weighted modulus of continuity $\Omega(f;\delta_{n}, \delta_{m}) $ (see [14]) defined by

24 $$ \Omega(f;\delta_{n}, \delta_{m}) = \sup _{(x,y)\in R_{+}^{2}} \sup_{|h_{1}|\leq \delta_{1}, |h_{2}|\leq\delta_{2}} \frac{|f(x+h_{1}, y+h_{2}) - f(x,y)|}{\rho (x,y) \rho(h1, h2)},\quad f\in C_{\rho}^{*} \bigl(R^{+}\bigr). $$

By the properties of weighted modulus of continuity $\Omega(f;\delta_{n}, \delta_{m}) $ (see [14], p.577) we have inequality

25 $$ \big|f(t,s) - f(x,y) \big| \leq8 \Omega(f;\delta_{n}, \delta_{m}) \bigl(1+x^{2}+y^{2}\bigr) g(t,x) g(s,y), $$

where $g(t,x)= ( ( 1+\frac{|t-x|}{\delta_{n}} ) (1+(t-x)^{2} ) )$ and $g(s,y)= ( ( 1+\frac {|s-y|}{\delta_{m}} ) (1+(s-y)^{2} ) )$. □

### Theorem 4.6

*For each* $f\in C_{\rho}^{*} (R^{+})$, *there exists a positive constant* *M*, *independent of* *n*, *m*, *such that*

$$\big\| C_{n,m}^{a}(f;x, y)-f(x, y) \big\| _{\rho^{3}}\leq M \Omega(f; \delta_{n}, \delta_{m}) $$

*for sufficiently large n*,*m*, *where* $\delta_{n} = {\frac{a_{n}}{n}} $ *and* $\delta_{m} = {\frac{v(a)}{n}}$.

### Proof

By the linearity and monotonicity of $C_{n,m}^{a}$ applied to inequality (24) we obtain

$$\begin{aligned} \big| C_{n,m}^{a}(f;x, y) - f(x, y)\big| \leq{}&8\frac{n}{a_{n}} c_{m} \Omega(f; \delta _{n}, \delta_{m}) \bigl(1+x^{2}+y^{2}\bigr) \\ &\times\sum_{k=0}^{n} p_{n,k} \biggl( \frac {x}{a_{n}} \biggr) \int_{\frac{k}{n}a_{n}}^{\frac{k+1}{n}a_{n}} g(t,x) \,dt \\ &\times\sum _{j=0}^{\infty}\Theta_{m,j}(b_{m}y,a) \int_{\frac {j}{c_{m}}}^{\frac{j+1}{c_{m}}} g(s,y)\, ds. \end{aligned}$$

Using the basic result obtained in [17]

$$g(t,x)\leq2 \bigl( 1+\delta_{n}^{2} \bigr) \bigl({1+\delta _{n}^{-4}(t-x)^{4}} \bigr) \quad\mbox{and}\quad g(s,y)\leq2 \bigl( 1+\delta_{m}^{2} \bigr) \bigl({1+\delta_{m}^{-4}(s-y)^{4}} \bigr), $$

we have

$$\begin{aligned} \big|C_{n,m}^{a} (f;x, y)-f(x, y)\big| \leq{}&8\Omega(f; \delta_{n}, \delta _{m}) \bigl(1+x^{2}+y^{2} \bigr) \times \biggl\{ 1+\frac{1}{\delta_{n}} {C_{n,m}^{a} \bigl( (e_{10}-x)^{4}; x, y \bigr)} \biggr\} \\ &\times \biggl\{ 1+\frac{1}{\delta_{m}} {C_{n,m}^{a} \bigl( (e_{01}-y)^{4}; x, y \bigr)} \biggr\} . \end{aligned}$$

Hence, by conditions (5) and (8) we immediately have

$$\begin{aligned} \big|C_{n,m}^{a} (f;x, y)-f(x, y)\big| \leq{}&8\Omega(f; \delta_{n}, \delta _{m}) \bigl(1+x^{2}+y^{2} \bigr) \times \Biggl\{ 1+\frac{1}{\delta_{n}} O \biggl( \frac {a_{n}}{n} \biggr) \Biggl(\sum_{i=0}^{4}x^{i} \Biggr) \Biggr\} \\ &\times \Biggl\{ 1+\frac{1}{\delta_{m}} \frac{\omega(a)}{c_{m}} \Biggl(\sum _{i=0}^{4}y^{i}\Biggr) \Biggr\} . \end{aligned}$$

Choosing $\delta_{n} = \frac{a_{n}}{n}$ and $\delta_{m} = {\frac{v(a)}{n}}$, there exists a positive constant *M*, independent of *n*, *m*, such that the following inequality is satisfied:

$$\big|C_{n,m}^{a} (f;x,y)-f(x,y)\big| \leq M\Omega(f; \delta_{n}, \delta _{m}) \bigl(1+x^{2}+y^{2} \bigr) \times \Biggl\{ 1+ \sum_{i=0}^{4}x^{i} \Biggr\} \times \Biggl\{ 1+ \sum_{i=0}^{4}y^{i} \Biggr\} . $$

For sufficiently large *n*, *m*, we obtain

$$\big\| C_{n,m}^{a}(f;x, y)-f(x, y) \big\| _{\rho^{3}}\leq M \Omega(f; \delta_{n}, \delta_{m}), $$

which implies the desired result. □

## Approximation in the space of Bögel continuous functions

In this section, we give a generalization of the operators defined in (3) for the B-continuous functions. First, we need to introduce a GBS operator related to bivariate Chlodowsky-Szasz-Kantorovich-Charlier-type operators and investigate some of its smoothness properties. The concepts of B-continuity and B-differentiability were initiated by Bögel [18, 19]. To provide uniform approximation of B-continuous functions, GBS operators are used. For the first time, the term GBS operators were introduced by Badea et al. [20, 21]. A well-known theorem for approximation of B-continuous and B-differentiable functions was presented and proved by Bögel et al. [18]. Recently, Agrawal and Ispir [6] established the degree of approximation for bivariate Chlodowsky-Szász-Charlier-type operators. In [22], GBS operators of Lupas-Durrmeyer type based on Polya distribution are defined. The degree of approximation is also discussed by means of the mixed modulus of smoothness and the mixed *K*-functional. Further, Agrawal and Sidharth [10] introduced the approximation of Bögel continuous functions by GBS operators and discussed the degree of approximation by means of the Lipschitz class of Bögel continuous functions, mixed modulus of smoothness, and the mixed *K*-functional. Significant contribution in the area of approximation theory are done by several researchers [12, 23–25]. Inspired by the above work, we propose the GBS operators with the operator defined by relation (3). Now, we recall some basic definitions and notation. The details can be found in [18, 19].

Let *I* and *J* be compact real intervals, and $A=I\times J$. For any $f:A\to R$ and any $(t,s), (x,y) \in A$, let $\triangle_{(t,s)} f(x,y)$ be the bivariate mixed difference operators defined as

$$\triangle_{(t,s)} f(x,y) = f(t,s)-f(t,y)-f(x,s)+f(x,y). $$

A function $f:A\to R$ is called a B-continuous (Bögel-continuous) at $(x,y) \in A $ if

$$\lim_{(t,s)\to(x,y)} \triangle_{(t,s)}f(x,y) = 0. $$

If *f* is B-continuous at any $(x,y) \in A$, then *f* is B-continuous on *A*. We denote by $C_{b}(A) = \{ f|f:A\to R, f \mbox{ B-bounded on } A\} $, the space of all B-continuous functions on *A*. A function $f:A\to R$ is called B-differentiable on $(x,y) \in A $ if the following limit exists and is finite:

$$\lim_{(t,s)\to(x,y)} \frac{\triangle_{(t,s)}f(x,y) }{(t-x)(s-y)} = D_{B} f(x,y)< \infty. $$

We denote by $D_{b}(A) = \{ f|f:A \to R, f \text{ B-differentiable on } A\} $ the space of all B-differentiable functions.

The function $f:A\to R$ is B-bounded on *D* if there exists $K>0$ such that $|\triangle_{(t,s)f(x,y)}|\leq K$ for all $(t,s), (x,y) \in A$. Here, if *A* is a compact subset, then each B-continuous function is a B-bounded function on *A*. We denote by $B_{b}(A)$ the space of all B-bounded functions on *A* equipped with the norm $\| f \|_{B} =\sup_{(x,y),(t,s) \in A} \vert \triangle_{(t,s)}f(x,y) \vert $.

To evaluate the approximation degree of a B-continuous function using linear positive operators, an important tool is the mixed modulus of continuity. Let $f\in B_{b}(I_{a_{n}})$. The mixed modulus of continuity of *f* is the function $\omega_{B}:[0,\infty)\times[0, \infty)\to R $ defined by

$$\omega_{B}(f;\delta_{1}, \delta_{2}) = \sup\bigl\{ \triangle_{(t,s)}f(x,y): |t-x|\leq\delta_{1}, |s-y| \leq \delta_{2} \bigr\} $$

for $(t,s), (x,y)\in A$.

For $I_{de} = [0,d]\times[0,e]$, let $C_{b}(I_{de})$ denote the space of all B-continuous functions on $I_{de}$, and let $C(I_{de})$ be the space of all ordinary continuous functions on $I_{de}$.

We define the GBS operators of the $C_{n,m}^{a}$ given by (3), for any $f\in C(I_{de})$ and $n,m \in N$, by

26 $$ S_{n,m}^{a} \bigl(f(t,s);x,y\bigr) = C_{n,m}^{a} \bigl(f(t,y)+f(x,s) - f(t,s);x,y\bigr) $$

for all $(x,y) \in I_{de}$.

More precisely, for any $f\in C(I_{de})$, the GBS operator of Chlodowsky-Szász-Kantorovich-Charlier operators is given by

$$\begin{aligned} S_{n,m}^{a}(f;x,y) = {}&\frac{n}{a_{n}} c_{m} \sum_{k=0}^{n} \sum _{j=0}^{\infty}p_{n,k} \biggl( \frac{x}{a_{n}} \biggr) \Pi_{m,j}(b_{m}y,a) \\ &\times \int_{\frac{j}{c_{m}}}^{\frac{j+1}{c_{m}}} \int_{\frac {k}{n}a_{n}}^{\frac{k+1}{n}a_{n}} \bigl(f(t,y)+f(x,s) - f(t,s);x,y \bigr)\,dt\,ds. \end{aligned}$$

### Theorem 5.1

*If* $f\in C_{b} (I_{de})$, *then for any* $(x,y) \in I_{de}$ *and any* $m,n\in N$, *we have*

$$\big|S_{m,n}^{a} \bigl(f(t,s);x,y\bigr) - f(x,y)\big| \leq4 \omega_{B} (f; \delta_{n}, \delta_{m}), $$

*where* $\delta_{n} = ( \rho(a) \frac{a_{n}}{n} )^{1/2}$ *and* $\delta_{m} = ( \frac{\varsigma(a)}{c_{m}} )^{1/2}$.

### Proof

By using the properties of $\omega_{B}$ we obtain

27 $$ \bigl\vert \triangle_{(x,y)} f(t,s) \bigr\vert \leq \omega_{B}\bigl(f;|t-x|, |s-y|\bigr) \leq \biggl( 1+ \frac{|t-x|}{\delta_{n}} \biggr) \biggl( 1+\frac {|s-y|}{\delta_{m}} \biggr) \omega_{B}(f; \delta_{n}, \delta_{m}) $$

for all $(x,y), (t,s) \in I_{de}$ and $\delta_{n}, \delta_{m} >0$. Hence, from the monotonicity and linearity of the operators $S_{n,m}^{a} ( f(t,s); x, y )$, using the Cauchy-Schwarz inequality, we get from (26) that

$$\begin{gathered} \big|S_{n,m}^{a} \bigl( f(t,s); x, y \bigr)-f(x, y)\big|\\ \quad\leq C_{n,m}^{a} \bigl( \big|\Delta_{(x, y)}f(t, s)\big|; x, y \bigr) \\ \quad\leq \biggl( C_{n,m}^{a} (e_{00}; x, y ) + \frac{1}{\delta_{n}} \bigl(C_{n,m}^{a} \bigl( (e_{10}-x)^{2}; x, y \bigr) \bigr)^{1/2} \\ \qquad{}+ \frac{1}{\delta_{m}} \bigl(C_{n,m}^{a} \bigl( (e_{01}-y)^{2}; x, y \bigr) \bigr)^{1/2} + \frac{1}{\delta_{n}} \bigl(C_{n,m}^{a} \bigl( (e_{10}-x)^{2}; x, y \bigr) \bigr)^{1/2} \\ \quad\quad{}\times \frac{1}{\delta_{m}} \bigl(C_{n,m}^{a} \bigl( (e_{01}-y)^{2}; x, y \bigr) \bigr)^{1/2} \biggr) \omega_{B}(f; \delta_{n}, \delta_{m}). \end{gathered}$$

Using inequalities (9) and (10), we have

$$\begin{gathered} \big| S_{n,m}^{a} \bigl( f(t,s); x, y \bigr)-f(x, y) \big| \\ \quad\leq \biggl\{ 1+\frac {1}{\delta_{n}} \biggl( \rho(a) \frac{a_{n}}{n} \biggr)^{1/2}+ \frac{1}{\delta _{m}} \biggl( \frac{\varsigma(a)}{c_{m}} \biggr)^{1/2}+ \frac{1}{\delta_{n} \delta_{m}} \biggl( \rho(a) \frac{a_{n}}{n} \biggr)^{1/2} \biggl( \frac {\varsigma(a)}{c_{m}} \biggr)^{1/2} \biggr\} , \end{gathered}$$

from which the desired result is immediate by choosing $\delta_{n}= ( \rho(a) \frac{a_{n}}{n} )^{1/2} $ and ${\delta_{m}}= ( \frac {\varsigma(a)}{c_{m}} )^{1/2}$. □

In the following, the approximation of Lipschitz class of B-continuous functions. For $0<\gamma\leq1$, let

$$\operatorname{Lip}_{L}\gamma= \bigl\{ f\in C(I_{a_{n}}): \big| \Delta_{(x,y)}f[t,s;x,y]\big| \leq L\|r-s\|^{\gamma} \bigr\} , $$

where $r=(u,v)$, $s=(x,y)\in I_{a_{n}}$, and $\| r-s\|= \{ (u-x)^{2}+(v-y)^{2} \}^{1/2}$ is the Euclidean norm, be the Lipschitz class of B-continuous functions. The next result gives the rate of convergence of the operator $S_{n,m}^{a} ( f(t,s); x, y )$ in terms of the Lipschitz class.

### Theorem 5.2

*If* $f \in \operatorname{Lip}_{L} \gamma$, *then for every* $(x,y)\in I_{de}$, *we have*

$$\bigl\vert S_{n,m}^{a} \bigl( f(t,s);x, y \bigr)-f(x, y) \bigr\vert \leq L \bigl\{ \delta_{n}(x)+\delta_{m}(y) \bigr\} ^{\gamma/2} $$

*for* $L>0$ *and* $\gamma\in(0,1]$.

### Proof

Using the definition of the operators $S_{n,m}^{a} ( f(t, s); x, y )$, we can write

$$\begin{aligned} S_{n,m}^{a} \bigl( f(t, s); x, y \bigr) &= C_{n,m}^{a} \bigl( f(x, s)+f(t, y)- f(t, s); x, y \bigr) \\ &= C_{n,m}^{a} \bigl( f(x, y)- \Delta_{(x,y)}f(t, s) ; x, y \bigr)\\& = f(x, y) C_{n,m}^{a} ( e_{00}; x, y ) - C_{n,m}^{a} \bigl(\Delta_{(x, y)} f(t, s); x, y \bigr). \end{aligned}$$

By the hypothesis we get

$$\bigl\vert S_{n,m}^{a} \bigl( f(t, s); x, y \bigr) - f(x, y) \bigr\vert \leq C_{n,m}^{a} \bigl( \bigl\vert \Delta_{(x,y)}f(t, s) \bigr\vert ; x, y \bigr) \leq L C_{n,m}^{a} \bigl(\| r-s\|^{\gamma}; x, y \bigr). $$

For $u_{1} = \frac{2}{\gamma}$ and $v_{1} = \frac{2}{2-\gamma}$, applying the Hölder inequality and Remark 2.3, we get

$$\begin{aligned} \big\vert S_{n,m}^{a} \bigl( f(t, s); x, y \bigr) -f(x, y) \big\vert &\leq L \bigl\{ C_{n, m}^{a} \bigl( \| r-s\|^{2}, x, y \bigr) \bigr\} ^{\gamma/2}\\ &\leq L \bigl\{ C_{n,m}^{a} \bigl( (u-x)^{2}, x,y \bigr) +C_{n,m}^{a} \bigl( (v-y)^{2}, x, y \bigr) \bigr\} ^{\gamma/2}, \end{aligned}$$

which leads us to the required result. □

### Theorem 5.3

*If* $f\in D_{b}(I_{de})$ *and* $D_{B}f\in B(I_{de})$, *then*, *for each* $(x,y)\in I_{de}$, *we get*

$$\begin{aligned} \big|{S}_{n,m}^{a}(f;x,y)-f(x,y)\big|\leq{}& C \bigl\{ 3 \|D_{B} f\|_{\infty}+2 \omega _{\mathrm{mixed}}(f; \delta_{n},\delta_{m})\sqrt{x^{2}+x} \sqrt{y^{2}+y+1} \bigr\} \delta_{n} \delta_{m} \\ &+ \bigl\{ \omega_{\mathrm{mixed}}(f;\delta_{n},\delta_{m}) \bigl(\delta_{m}\sqrt {x^{4}+x^{3}+x^{2}+x} \sqrt{y^{2}+y+1} \\ &+\delta_{n}\sqrt{y^{4}+y^{3}+y^{2}+y+1} \sqrt{x^{2}+x} \bigr) \bigr\} , \end{aligned}$$

*where* $\delta_{n}=\sqrt{\frac{a_{n}}{n}}$, $\delta_{m}=\sqrt{\frac{\eta (a)}{c_{m}}}$, $\eta(a)=\max \{\tau(a),\omega(a) \}$, *and* *C* *is a constant depending on* *n*, *m* *only*.

### Proof

By our hypothesis,

$$ \Delta_{(x,y)}f(t,s)=(t-x) (s-y)D_{B} f(\alpha,\beta), \quad\text{with } x< \alpha< t ; y< \beta< s. $$

Clearly,

$$ D_{B} f(\alpha,\beta)=\Delta_{(x,y)}D_{B} f( \alpha,\beta)+D_{B} f(\alpha ,y)+D_{B} f(x, \beta)-D_{B} f(x,y). $$

Since $D_{B} f\in B(I_{de})$, from the above equalities we have

28 $$ \begin{aligned}[b] &\big|S_{n,m}^{a}\bigl(\Delta_{(x,y)}f(t,s);x,y \bigr)\big|\\ &\quad=\big|S_{n,m}^{a}\bigl((t-x) (s-y)D_{B} f( \alpha,\beta);x,y\bigr)\big| \\ &\quad\leq S_{n,m}^{a}\bigl(|t-x||s-y|\big|\Delta_{(x,y)}D_{B} f(\alpha,\beta )\big|;x,y\bigr) \\ &\qquad{}+S_{n,m}^{a}\bigl(|t-x||s-y|\bigl(\big|D_{B} f( \alpha,y)\big|+\big|D_{B} f(x,\beta)\big|+\big|D_{B} f(x,y)\big|\bigr);x,y\bigr) \\ &\quad\leq S_{n,m}^{a}\bigl(|t-x||s-y|\omega_{\mathrm{mixed}}\bigl(D_{B} f;|\alpha-x|,|\beta -y|\bigr);x,y\bigr) \\ &\qquad{}+3 \|D_{B} f\|_{\infty} S_{n,m}^{a}\bigl(|t-x||s-y|;x,y\bigr). \end{aligned}$$

By the properties of mixed modulus of smoothness $\omega_{\mathrm{mixed}}$ we can write

29 $$ \begin{aligned}[b]\omega_{\mathrm{mixed}}\bigl(D_{B} f;|\alpha-x|,|\beta-y|\bigr)& \leq\omega_{\mathrm{mixed}}\bigl(D_{B} f;|t-x|,|s-y|\bigr) \\ &\leq\bigl(1+\delta^{-1}_{n}|t-x|\bigr) \bigl(1+ \delta^{-1}_{m}|s-y|\bigr) \omega_{\mathrm{mixed}}(D_{B} f;\delta_{n},\delta_{m}). \end{aligned}$$

Combining (28) and (29) and using the Cauchy-Schwarz inequality, we find

30 $$\begin{aligned} \big|S_{n,m}^{a}(f;x,y)-f(x,y)\big| =&\big|S_{n,m}^{a} \Delta _{(x,y)}f(t,s);x,y\big| \\ \leq& 3\|D_{B} f\|_{\infty}\sqrt{S_{n,m}^{a} \bigl((t-x)^{2}(s-y)^{2};x,y\bigr)} \\ &{}+ \bigl(S_{n,m}^{a}\bigl(|t-x||s-y|;x,y\bigr) +\delta^{-1}_{n} S_{n,m}^{a} \bigl((t-x)^{2}|s-y|;x,y\bigr) \\ &{}+\delta^{-1}_{m} S_{n,m}^{a}\bigl(|t-x|(s-y)^{2};x,y\bigr) \\ &{} +\delta^{-1}_{n}\delta^{-1}_{m} S_{n,m}^{a}\bigl((t-x)^{2}(s-y)^{2};x,y \bigr) \bigr)\omega_{\mathrm{mixed}}(D_{B} f;\delta_{n}, \delta_{m}) \\ \leq&3\|D_{B} f\|_{\infty}\sqrt{S_{n,m}^{a} \bigl((t-x)^{2}(s-y)^{2};x,y\bigr)} \\ &{}+ \Bigl(\sqrt {S_{n,m}^{a}\bigl((t-x)^{2}(s-y)^{2};x,y \bigr)} \\ & {}+\delta^{-1}_{n}\sqrt{S_{n,m}^{a} \bigl((t-x)^{4}(s-y)^{2};x,y\bigr)} \\ &{}+\delta ^{-1}_{m}\sqrt{S_{n,m}^{a} \bigl((t-x)^{2}(s-y)^{4};x,y\bigr)} \\ & {}+\delta^{-1}_{n}\delta^{-1}_{m} S_{n,m}^{a}\bigl((t-x)^{2}(s-y)^{2};x,y \bigr) \Bigr)\omega_{\mathrm{mixed}}(D_{B} f;\delta_{n}, \delta_{m}). \end{aligned}$$

For $(x,y),(t,s)\in I_{de}$ and $i,j\in\{1,2\}$, we have

31 $$ S_{n,m}^{a}\bigl((t-x)^{2i}(s-y)^{2j};x,y \bigr)={_{x} B_{n}} \bigl((t-x)^{2i};x \bigr) {_{y} P^{*}_{m}} \bigl( (s-y)^{2j};y \bigr). $$

Since, by Remark 2.3,

$$ \begin{gathered}{_{x} B_{n}} \bigl((t-x)^{2};x \bigr)=O \biggl( \frac{a_{n}}{n} \biggr) \bigl(x^{2}+x\bigr), \\ {_{x} B_{n}} \bigl((t-x)^{4};x \bigr)=O \biggl( \frac{a_{n}}{n} \biggr) \bigl(x^{4}+x^{3}+x^{2}+x \bigr), \\ {_{y} P^{*}_{m}} \bigl( (s-y)^{2};y \bigr)\leq \frac{\tau(a)}{c_{m}}\bigl(y^{2}+y+1\bigr), \\ {_{y} P^{*}_{m}} \bigl( (s-y)^{4};y \bigr)\leq \frac{\omega(a)}{c_{m}}\bigl(y^{4}+y^{3}+y^{2}+y+1 \bigr), \end{gathered}$$

combining (30) and (31) and choosing $\delta_{n}=\sqrt{\frac {a_{n}}{n}}$, $\delta_{m}=\sqrt{\frac{\eta(a)}{c_{m}}}$, and $\eta(a)=\max(\tau (a),\omega(a))$, we get the required result. □

## Conclussion

The purpose of this paper is to provide a better error estimation of convergence by modification of Szász operators. We have defined a Szasz-Kantorovich-Chlodowsky generalization of these modified operators by using Charlier polynomials. This type of modification enables better error estimation for a certain function in comparison to the Szász-Kantorovich-Chlodowsky operators and Szasz-Chlodowsky-type operators based on Charlier polynomials. We obtained some approximation results via the well-known Korovkin-type theorem. We have also calculated the rate of convergence of operators by means of Peetre’s K-functional and partial moduli of continuity. Lastly, we discussed the degree of approximation for Bögel continuous and Bögel differentiable functions by means of the Lipschitz class and mixed modulus of smoothness.
